# Progressive tracking: a novel procedure to facilitate manual digitization of videos

**DOI:** 10.1242/bio.055962

**Published:** 2020-11-06

**Authors:** Maja Mielke, Peter Aerts, Chris Van Ginneken, Sam Van Wassenbergh, Falk Mielke

**Affiliations:** 1 Laboratory of Functional Morphology, Department of Biology, Faculty of Sciences, University of Antwerp, 2610 Wilrijk, Belgium; 2 Laboratory of Applied Veterinary Morphology, Department of Veterinary Sciences, Faculty of Biomedical, Pharmaceutical and Veterinary Sciences, University of Antwerp, 2610 Wilrijk, Belgium

**Keywords:** Videography, Point digitization, Tracking, Kinematics

## Abstract

Digitization of video recordings often requires the laborious procedure of manually clicking points of interest on individual video frames. Here, we present progressive tracking, a procedure that facilitates manual digitization of markerless videos. In contrast to existing software, it allows the user to follow points of interest with a cursor in the progressing video, without the need to click. To compare the performance of progressive tracking with the conventional frame-wise tracking, we quantified speed and accuracy of both methods, testing two different input devices (mouse and stylus pen). We show that progressive tracking can be twice as fast as frame-wise tracking while maintaining accuracy, given that playback speed is controlled. Using a stylus pen can increase frame-wise tracking speed. The complementary application of the progressive and frame-wise mode is exemplified on a realistic video recording. This study reveals that progressive tracking can vastly facilitate video analysis in experimental research.

## Abstract (precis)

**Summary:** Progressive tracking is a novel procedure for manual point digitization in markerless videos. It allows for tracking points efficiently without the need to click all individual frames.

## INTRODUCTION

Video recordings are of high relevance for scientific research in different fields. As technology advances, researchers make use of videography with increasingly high resolution and frame rate. Usually, analysis of such video data requires digitization of point movement (‘tracking’), a procedure that can get highly labor-intensive. To facilitate point digitization, several solutions for the automated tracking of moving objects are available, e.g. XMALab (Knörlein et al., 2016), Tracker (Brown and Cox, 2009), VideoTrack (ViewPoint, France), or the MATLAB applications XrayProject (Brainerd et al., 2010), DLTdv (Hedrick, 2008), and BEEtag (Crall et al., 2015). These mostly rely on traceable markers that can be easily recognized by computer algorithms.

However, manual tracking is often still necessary, e.g. because markers cannot be applied. In field research, for example, animals usually cannot be captured and tagged before filming (e.g. Druelle et al., 2020). The implantation of internal markers for x-ray recordings is not always possible. Some automated video digitization procedures require the manual generation of a training set. Others might require *post hoc* inspection and correction of episodes in which the algorithm produced inaccurate results. These are reasons why it is often still necessary to manually digitize markerless videos, i.e. to click the points of interest (POI) on the individual frames of the video (termed frame-wise tracking herein). Frame-wise tracking is the standard procedure for manual (i.e. non-automated) tracking in existing software such as XMALab (Knörlein et al., 2016), Tracker (Brown and Cox, 2009), and DLTdv (Hedrick, 2008).

Unfortunately, this frame-wise manual tracking can become highly labor-intensive. A high number of video frames magnifies the work load, making digitization time consuming and potentially even physically harmful (shoulder pain, neck pain, carpal tunnel syndrome, or other musculoskeletal symptoms; Karlqvist et al., 1996; Fagarasanu and Kumar, 2003). Furthermore, it may be desired to digitize POIs several times to average data, which increases the workload even more and easily results in clicking POIs on tens of thousands of images. Thus, it is desirable to develop solutions that facilitate manual tracking.

Here, we introduce ‘progressive tracking’, a procedure involving manual point digitization without the need to click. Instead of tracking points frame by frame, the user follows a POI with the cursor while the video is played at a flexible playback speed. As the video progresses, the cursor position is saved for each frame. As we demonstrate, the progressive tracking method requires software features which enable ad hoc adjustment of settings such as playback speed to retrieve accurate results despite variable POI movement. We provide such a tool for demonstration (supplementary file S1), yet our analysis herein is about the procedure, not the particular software tool. Progressive tracking has a high potential to make manual analysis of markerless videos faster and ergonomically more comfortable and has already been applied for scientific research by one of the authors (Mielke et al., 2020). However, the performance of progressive and frame-wise tracking has not been quantified with regard to speed and accuracy. In the first part of this study, we compare tracking speed and tracking accuracy of both methods applied on an artificial test data set. We introduce the additional option of using a stylus pen on a digital drawing display as input device instead of a computer mouse. We test how using such a pen affects the performance of frame-wise or progressive tracking.

Frame-wise and progressive tracking are non-exclusive: in fact, the supplemented software tool implements both frame-wise tracking (as applied in existing software) and progressive tracking, allowing the user to switch between these strategies at any time. In the second part of our study, we exemplify the complementary application of progressive and frame-wise tracking on a video recording from our own research in order to demonstrate a realistic point digitization scenario. We discuss different measures that should be taken into account regarding video preparation and tracking process.

## RESULTS

### Test data: videos of a moving point

#### Tracking speed is higher with progressive mode

**Fig. 1.**
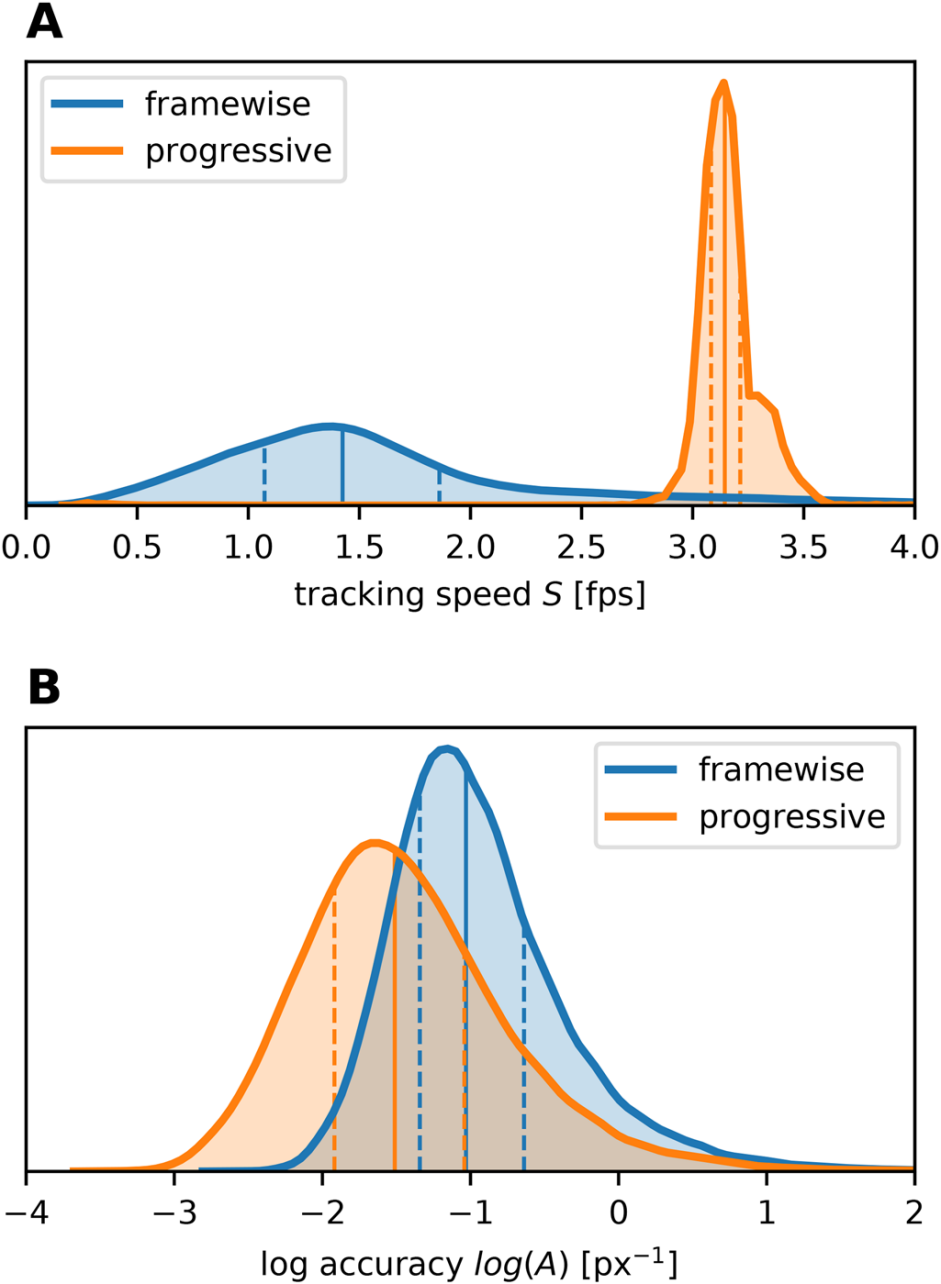
**Speed and accuracy of frame-wise and progressive tracking.** Progressive tracking is (A) much faster, but (B) less precise compared to frame-wise tracking. Note that accuracy *A* is logarithmized. The curves show distributions of the pooled data with quartiles (*Q*_50%_: solid line; *Q*_25%_, *Q*_75%_: dashed lines). Sample sizes: *N*_*fw*_=39,945, *N*_*pr*_=39,905 (ten videos, four replications per mode).

**Fig. 2.**
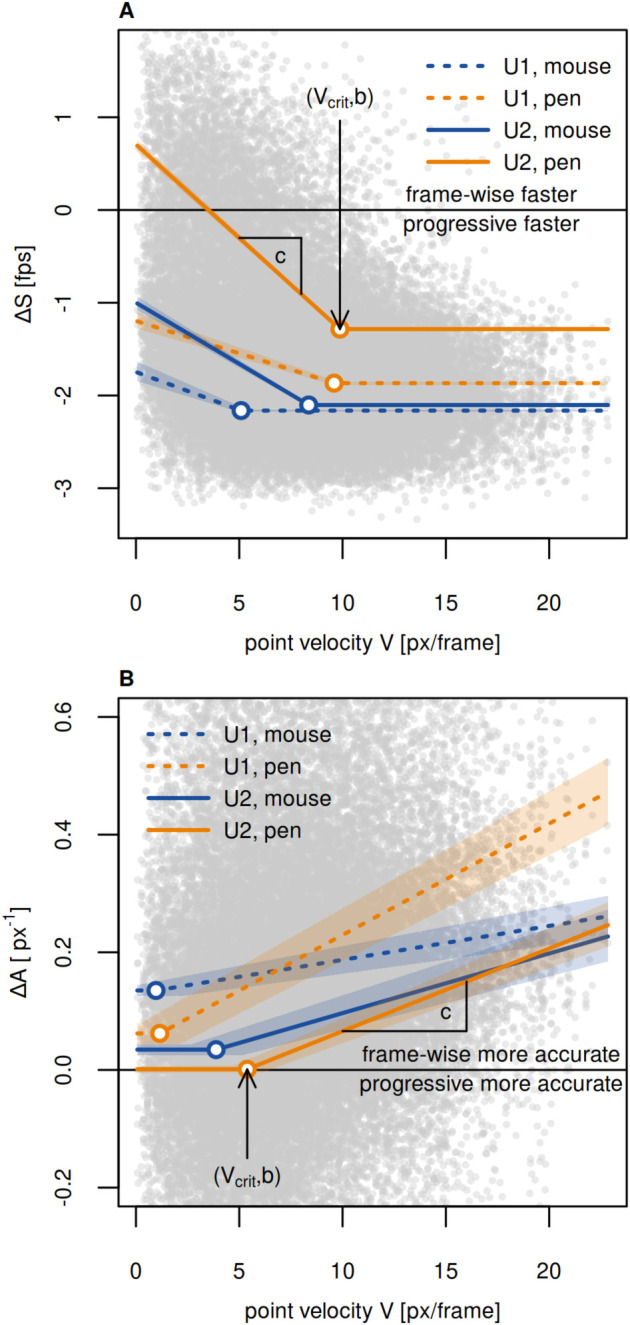
**Δ*S* and Δ*A* in relation to point velocity *V*.** (A) Δ*S* is mostly negative, indicating that frame-wise tracking is slower than progressive tracking. The difference in tracking speed is lower for low point velocities. (B) Δ*A* is mostly positive, indicating that frame-wise tracking is more precise. The difference in tracking accuracy increases with point velocity, because we did not restrict playback speed for high *V*. The gray shade in the background represents the pooled raw data. Colored lines indicate the relationship of Δ*S* or Δ*A* with point velocity for both users (dashed versus solid lines) and both tracking devices (orange versus blue lines). Colored shading indicates the 89% probability interval.

In order to assess to what extent progressive tracking is faster than frame-wise tracking, we calculated the speed *S* for both methods (Eqn 1). With progressive tracking (using a fixed inter-frame interval and correcting tracking if required), we achieved a median speed of *S*_*pr*_=3.14 fps (Fig. 1A). Frame-wise tracking (median *S*_*fw*_=1.42 fps) hardly ever reached this value and was almost always outperformed by progressive mode (Fig. 1A). Only for low point velocities, frame-wise tracking was occasionally faster than the progressive tracking speed tested herein (Fig. 2A). As tracking speed of progressive tracking was constrained by fixed playback speed, this is only due to faster frame-wise tracking. For the range between 0 and the critical point velocity *V*_*crit*_, speed difference Δ*S* (Eqn 3) and point velocity *V* are negatively correlated (slope *c*), i.e. the higher *V*, the lower *S*_*fw*_. For point velocities greater than *V*_*crit*_, tracking speed difference is constant (Δ*S*=*b*). Note that the values for *c*, *V*_*crit*_, and *b* vary with user and tracking device (Fig. 2A, Table 1). We conclude that the progressive tracking method can considerably speed up manual digitization of videos.

**Table 1.**
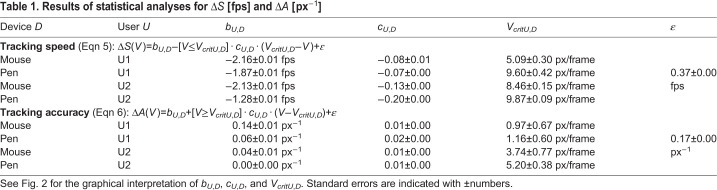
**Results of statistical analyses for** Δ*S* **[fps] and** Δ*A* **[px**^−1^**]**

#### Frame-wise tracking is more accurate than progressive tracking

**Fig. 3.**
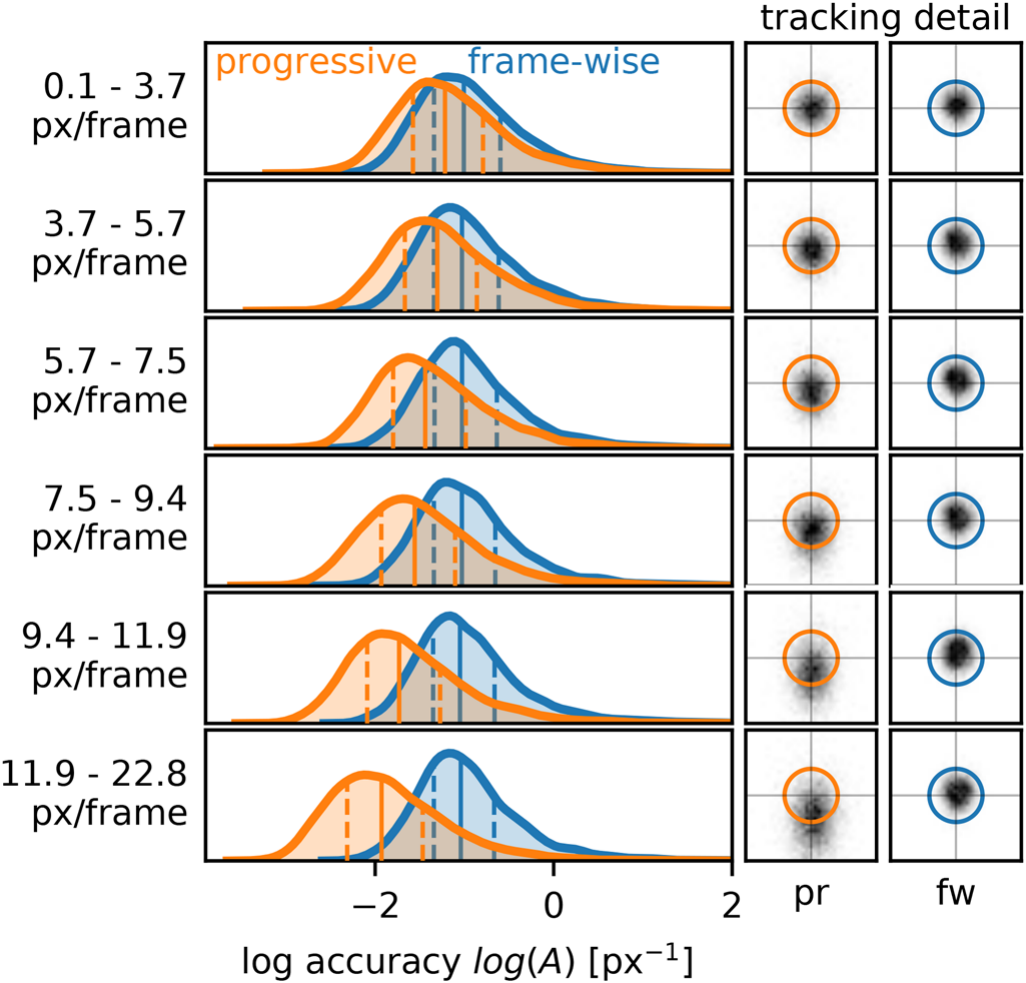
**Tracking accuracy against point velocity.** The tracking accuracy of frame-wise tracking is independent of point velocity. For low point velocities, *A*_*pr*_ approximately matches *A*_*fw*_. Given a fixed playback speed as used herein, *A*_*pr*_ decreases with increasing point velocity. The curves show distributions of the pooled data with quartiles (*Q*_50%_: solid line; *Q*_25%_, *Q*_75%_: dashed lines). Sample size per distribution: *N*=6,649±4 (ten videos, four replications). Note that accuracy *A* is logarithmized. In the detail plots (32×32 px), circles represent the point, with tracked positions indicated in gray. The positive y-axis corresponds to the direction of point velocity, which reveals that the track position lags behind the true position on higher *V*-values.

In order to evaluate the accuracy of progressive and frame-wise mode, we measured the distance *d* to the reference point to calculate tracking accuracy *A* (Eqn 2). Frame-wise tracking (mean *A*_*fw*_=0.50 px^–1^) was overall more accurate than fixed speed progressive tracking (mean *A*_*pr*_=0.32 px^–1^; Fig. 1B). The mean distance *d* differed by 2.24 px between frame-wise and progressive mode (*d*_*fw*_=2.96 px, *d*_*pr*_=5.20 px, the latter still being within the 6.5 px radius range of the tracked point). However, for low point velocities *V*, the accuracy of progressive tracking approximately matched that of frame-wise mode (Fig. 3). For high point velocities, *A*_*pr*_ declined with increasing *V* due to the fixed playback speed, whereas *A*_*fw*_ remained constant (Figs 2B and 3). The offsets *d*_*pr*_ for high *V* are biased by the direction of point movement (i.e. point following is delayed, see Fig. 3), which confirms the velocity-dependence. The difference Δ*A* (Eqn 4) in tracking accuracy is constant (Δ*A*=*b*) for low point velocities between 0 and *V*_*crit*_, indicating that increasing *V* does not reduce tracking accuracy within this range (Fig. 2B). For higher *V*, however, Δ*A* and *V* are positively correlated (slope *c*). As observed for Δ*S*, the parameters *b*, *c*, and *V*_*crit*_ vary with user and tracking device (Fig. 2B and Table 1). We conclude that the accuracy of progressive tracking can match that of frame-wise mode, given that the point velocity remains below a critical value.

#### Using a stylus pen increases frame-wise tracking speed

In order to test how using a stylus pen affects tracking performance, we analyzed how Δ*S* and Δ*A* differ between mouse- and pen-tracking. We found that Δ*S* was higher for pen-tracking (Fig. 2A), indicating that using a pen increased *S*_*fw*_ (*S*_*pr*_ was constrained by the fixed inter-frame interval). Mean frame-wise pen-tracking (*S*_*fw*,*pen*_=1.9 fps) was more than 50% faster than mouse-tracking (*S*_*fw*,*mouse*_=1.2 fps). For low point velocities, frame-wise pen-tracking even outperformed progressive tracking speed as applied herein (Fig. 2A). We did not find a clear evidence for a correlation of Δ*A* and input device (Fig. 2B). We conclude that using a stylus pen does not necessarily improve accuracy, but can accelerate manual digitization of videos when frame-wise tracking is applied.

### Real data: video of piglet locomotion

#### Progressive mode increases tracking speed

**Fig. 4.**
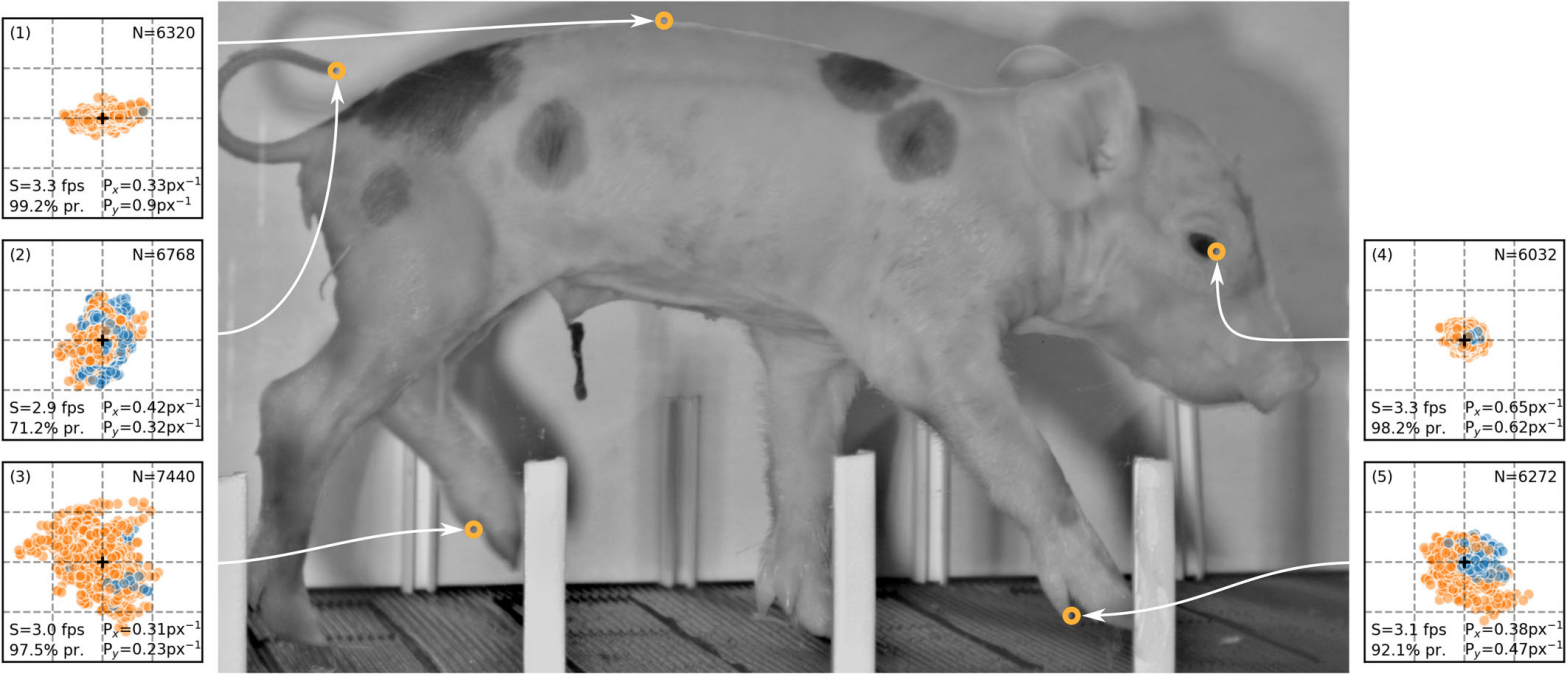
**Piglet video tracking.** Orange: progressive, blue: frame-wise. Texts indicate tracking speed *S*, ratio of progressive tracking used, tracking precision *P*, and sample size *N*. Data are centered around their total mean (+), grid lines mark 15 px ranges. Each point has been tracked 16 times. See Movie 1 for the whole video with tracked points.

To demonstrate the complementary application of progressive and frame-wise tracking, we selected a video of a locomoting piglet and digitized movement of five POIs, 16 times each. We aimed at intuitively optimizing both tracking speed (using progressive mode where possible) and tracking precision (using frame-wise mode where necessary). For all points, mostly progressive mode was used (71.2–99.2%, Fig. 4). Consequently, a speed of 2.9–3.3 fps was achieved, which is more than twice as fast as would be expected for pure frame-wise tracking. Frame-wise mode was mainly used during rapid/unpredictable movement and weak contrast, which both mainly occurred at the tail tip and resulted in the lowest average tracking speed (*S*_*tail*_=2.9 fps) for this point. Progressive mode was mainly used whenever the point was well-defined and moved with a constant and/or predictable velocity. During slow point movement, playback speed could be increased to more than 5.5 fps (maximal speed is constrained by computer hardware). We conclude that the complementary application of progressive and frame-wise mode can considerably enhance tracking speed in manual digitization of videos.

#### Tracking precision is affected by POI characteristics

To assess tracking precision, we calculated *P*_*x*_ and *P*_*y*_ (Eqn 7) of each POI, pooling all 16 trials and all video frames together. Precision highly depended on the appearance and movement profile of the point (Fig. 4; Movie 1). The eye (4), for example, was well defined, moved with constant velocity and was thus tracked with high and uniform precision (*P*_*x*_=0.65 px^−1^, *P*_*y*_=0.62 px^−1^). The vertebra (1), however, being much less defined in x-direction than in y-direction, displayed an unequal tracking precision for the two coordinates (*P*_*x*_=0.33 px^−1^, *P*_*y*_=0.9 px^−1^). The accessory hoof tips (3, 5) displayed motion blur during the swing phase and were occasionally hard to detect due to lack of contrast, which reduced tracking precision (0.23–0.47 px^−1^). The tail tip (2) was moving erratically and had poor contrast when being in front of the body. Consequently, the precision was rather low (*P*_*x*_=0.42 px^−1^, *P*_*y*_=0.32 px^−1^). We conclude that tracking precision is considerably affected by the appearance and movement of the POI and is mainly supported by a high contrast and constant movement profile.

## DISCUSSION

In this study, we showed that progressive tracking is faster but, above a critical point velocity, less precise than frame-wise tracking. Furthermore, we observed that using a stylus pen as input device can increase the speed of frame-wise tracking. Digitization of a real video revealed that using mostly progressive tracking can double the speed compared to pure frame-wise tracking. We furthermore showed that the precision depends on appearance and movement profile of the digitized points, highlighting the importance of contrast enhancement in video data.

Independent of whether digitization is performed manually or via automated methods, image contrast is key for good results. Our realistic test case provides evidence that low contrast (e.g. due to insufficient lighting, non-optimal exposure, and motion blur) can be a critical impairment. Such non-optimal point detectability reduces the precision even for frame-wise mode (see Fig. 4, point 2). To overcome this, repeated tracking should be applied in order to average trials, which improves data reliability. In our test data analysis (point videos), we presented replicates individually, but averaging the four trials of progressive tracking would have increased the accuracy by 20.8%. This implies that repeated tracking (which is facilitated by fast progressive mode) and averaging reduces data noise that results from weak contrast.

In realistic applications, the user can flexibly adjust zoom and playback speed, as we did when digitizing the piglet video. Note that these adjustments are ad hoc, based on prior experience of the user, as opposed to fixing general settings for a certain type of analysis. Such adjustments have several consequences: (1) by increasing the playback speed, one can in theory track hundreds of frames per second if the point of interest hardly moves for a certain section within the video (note that apparent movement is inversely correlated with video recording frame rate, and progressive tracking is particularly useful for high speed recordings). We consider this the main advantage of progressive tracking, as such hardly moving points can be digitized with high accuracy and high speed. (2) By decreasing the playback speed, one can retain tracking accuracy during episodes of fast point movement. Reducing the playback speed increases *V*_*crit*_, and thus widens the range of point velocities in which progressive tracking is approximately as accurate as frame-wise mode. (3) By zooming in, one can generally increase tracking accuracy. However, zooming in also increases the effective velocity of the point in the progressing video. Thus, accordingly, (4) by zooming out, one can slow down the point of interest. To sum up, the user can and should flexibly adjust and balance the zoom factor and playback speed according to the current point velocity. Tracking software should be designed to facilitate this crucial flexibility requirement, for example by shortcuts and options for personalization.

There are multiple factors which affect the critical point velocity *V*_*crit*_ at which tracking accuracy of the progressive method declines compared to the frame-wise mode. Not only user and input device are critical (as shown herein), but also frame rate, progression speed, zoom rate, and video resolution (which all were kept constant in our analysis). Thus, it is not possible to give a general advice for an ideal play back speed during progressive tracking. However, we do advise to take the time to familiarize with the progressive tracking method before applying it for actual data analysis. Thorough visual examination of the tracking results will give an idea of the adequacy of tracking performance. By getting practical experience, users will automatically improve overall tracking accuracy/precision and get a feeling for the appropriate tracking settings, which are specific to their videographic setup. Users who need high accuracy are advised to track repeatedly and confirm *ex post* that the standard deviation at each frame is not biased by the direction of instantaneous point velocity.

As a side note, we would like to mention the implications of the introduced methods on workplace ergonomics. Using progressive tracking would allow researchers to omit thousands of mouse clicks every day, which can prevent musculoskeletal symptoms such as muscle pain or the carpal tunnel syndrome (Karlqvist et al., 1996; Fagarasanu and Kumar, 2003). The suggested procedure is thus not only faster, but also ergonomically more convenient. Independent of the tracking mode, using a pen-shaped input device can be beneficial for work performance and muscular load (Kotani and Horii, 2003; Ullman et al., 2003). Users without prior experience might need a short period to familiarize with a pen as input device, but soon achieve better performance than with a computer mouse and feel comfortable with pen handling (Kotani and Horii, 2003). This should be taken into account when selecting an input device for video digitization.

As all variants of point digitization methods, progressive tracking has limitations. When intentionally tracking at a fixed speed that was inappropriate for high point velocities, cursor movement of the user lagged behind the true point movement (Fig. 3), which is plausible. This emphasizes that supercritical velocities should generally be avoided by limiting the playback speed, thereby increasing *V*_*crit*_. Repeated tracking, besides increasing precision by averaging (see above), should reveal critical periods by a bias in the standard deviation components that are parallel and perpendicular to the momentary point displacement. This emphasizes the importance of repeated tracking, which benefits from the tracking speed advantage of progressive tracking.

The tracking results of the piglet video reveal that fast moving POIs are tracked with lower precision. The hoof tips (points 3, 5) show some outliers in the data obtained via progressive tracking. We consider the weak contrast due to motion blur as the main reason for this loss in precision. However, we want to stress that the video introduced an additional obstacle, as the hoof tips were partially hidden behind columns next to the runway or behind other limbs (see Movie 1). We intentionally included these frames in order to test how progressive tracking can help to interpolate the hidden points. This obstacle probably lowered overall tracking precision in our analysis. Nevertheless, we consider progressive tracking an adequate method to interpolate such gaps, as it allows for pursuing a trajectory in a more uniform way than frame-wise mode.

Progressive tracking is a method particularly designed for digitization of high frame rate videos. If the frame rate is low compared to the movement of a POI, frame-to-frame distances will be high, which complicates smooth progressive tracking even at a low playback speed. Thus, we recommend to switch to frame-wise tracking whenever a poor frame rate to displacement ratio results in a jerky point progression.

We conceived and tested progressive tracking with a clear focus on manual, markerless tracking. However, to some extent, it can also be useful for digitization workflows that involve automated methods. First, automated methods usually require a training set, and unless frames are randomized for training set generation, the efficiency increase we measured will also apply. Second, *ex post* inspection and correction of automated methods can be facilitated if software tools feature playback at adjustable speed.

In conclusion, our study indicates that progressive tracking is a valuable tool to increase the efficiency of manual point digitization in markerless videos, which can reduce the dependence on marker implantation in scientific research. Furthermore, progressive tracking improves the physical comfort of video analysis, potentially preventing musculoskeletal disorders. For those who still rely on frame-wise tracking for manual digitization of videos, we propose the option of using a stylus pen with a digital drawing display. This device increases frame-wise tracking efficiency and will feel more natural than mouse clicking for many applicants. Thus, progressive tracking and the use of a stylus pen for point digitization represent promising options to vastly facilitate video analysis in scientific research.

## MATERIALS AND METHODS

### Test data: videos of a moving point

With a python script (supplementary file S2), we generated ten videos (1000 frames each) of a moving point, moving randomly with varying 2D acceleration, resulting in a point velocity range of 0 to 22.8 px/frame. The point was 13 px in diameter and slightly blurred at the edges. Point movement was digitized with both frame-wise and progressive tracking mode. In order to test whether the use of a stylus pen on a digital drawing display affects tracking performance, we also tested that alternative input device for both frame-wise and progressive mode. This resulted in four different methods all together (two modes×two devices). Tracking was conducted by two test persons (M.M. and F.M.) independently with the custom built Progressive Tracker (supplementary file S1). Each of the ten videos was digitized with all of the four methods (thus 40 trials per test person, in total 80,000 frames). To preclude potential learning effects, we randomized video order and switched both the video and the method after each trial.

For progressive tracking, we fixed the playback speed by setting the computational pause between frames to 0.1 s, thus fixing the inter-frame interval. The actual inter-frame interval is comprised by the pause and processing time. Hence, there is a maximum tracking speed which is determined by the computer hardware in relation to video data size. In our case, the final playback speed was approximately 3.22 fps. Normally, flexible adjustment of the playback speed is possible while tracking (e.g. to improve accuracy during sequences of high point velocity). However, we intentionally wanted to test how progressive tracking performs in terms of accuracy if we use a challenging playback speed. Furthermore, a fixed playback speed allowed us to determine the critical point velocity *V*_*crit*_ at which accuracy of progressive tracking declines compared to the frame-wise mode. Still, in order to maintain best possible accuracy, we did allow for correction of mistakes by going back in time and repeat digitizing with the fixed inter-frame interval. All videos were tracked using full screen view, without zooming in or out. The used hardware was constant for all videos and both test persons. A conventional computer mouse (Logitech M500) was used for mouse-tracking, an artist drawing display (XP-PEN Artist22E Pro) served as monitor, a stylus pen (XP-PEN P02S stylus) applied to the drawing display was used for pen-tracking.

To quantitatively characterize and compare the methods, we calculated tracking speed *S* and tracking accuracy *A* as follows:(1)
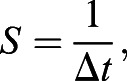
with Δ*t* being the time interval between two consecutive frames.(2)
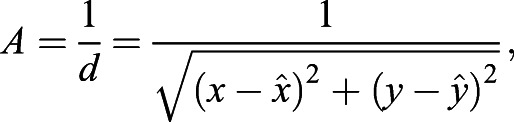
where *d* is the Euclidean distance between the center of the moving point 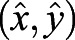 and the tracked position (*x*, *y*). According to these definitions, high values of *S* and *A* reflect a faster and more accurate tracking performance, respectively.

In further analyses, we wanted to assess how speed *S* and accuracy *A* differ between tracking modes and how this difference is related to point velocity *V*. Because we tracked identical frames with all methods, our measurements are not statistically independent. Therefore, we used the differences Δ*S* and Δ*A* of each frame *f* as outcome variable.(3)
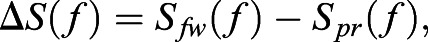
(4)
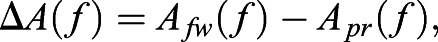


with *fw* and *pr* indicating the tracking modes ‘frame-wise’ and ‘progressive’, respectively.

Detailed raw data of the point video tracking are provided in the supplements (Tables S1 and S2).

### Statistical analysis with probabilistic modeling

We used probabilistic modeling in R (R Core Team, 2020) and Python (Python Software Foundation, 2020) to fit a statistical model to our data. The model aims at inferring how Δ*S*=*S*_*fw*_−*S*_*pr*_ and Δ*A*=*A*_*fw*_−*A*_*pr*_ (outcome variables) are related to point velocity *V* (predictor variable), taking both tracking device *D* (mouse or pen) and user *U* (U1 or U2) into account. To control for computational artifacts, only frames with finite *S*>0.2 fps were included in the analysis.

Given the fixed playback speed for progressive tracking in our test case, changes in Δ*S* are mainly due to variation in *S*_*fw*_. Frame-wise tracking can be fast for low point velocities, because the cursor hardly has to be moved between clicks, but reaches a constant minimum for *V*>*V*_*crit*_. Thus, we assumed Δ*S* to be constant for *V*>*V*_*crit*_ and to be linearly related to *V* for *V*≤*Vcrit* (Eqn 5, Fig. 2A).

Given the match of tracking accuracy for progressive and frame-wise mode for low point velocities, we assumed that Δ*A* is not affected by *V* if *V*<*V*_*crit*_. For *V*≥*V*_*crit*_, we assumed a linear relationship between Δ*A* and *V* (Eqn 6, Fig. 2B).(5)
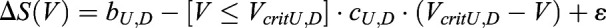
(6)
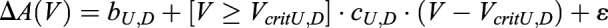


Therein, [*V*…*V*_*critU*,*D*_] is a boolean vector, being 1 or 0 depending on the expression being true or false, respectively. The parameters *b* (intercept), *c* (slope), and *V*_*crit*_ depend on user *U* and device *D*, which is indicated by the subscripts. The standard deviations of the fitted distributions are represented by *ε*. The models described above were favored by WAIC (Watanabe-Akaike information criterion) and LOO (leave-one-out cross validation) model comparison to models that left out either of the parameters *V*_*crit*_, *U*, or *D*.

The models were fit to the data using Markov chain Monte Carlo (MCMC) sampling, applied with the ulam() function of the rethinking R package version 2.01 (R Core Team, 2020; McElreath, 2020) and with PyMC3 in the Python programming language (Salvatier et al., 2016; Python Software Foundation, 2020). The R and Python code for the statistical analysis are independent and are both provided in the Supplemental Material (files S3 and S4).

### Real data: video of piglet locomotion

In order to demonstrate the complementary application of progressive and frame-wise tracking on realistic data, both test persons digitized five points on a video (800 frames) of piglet locomotion. We selected points that differ in their appearance and movement profile (Fig. 4 and Movie 1). Each of the two test persons tracked each of the five points eight times (in total 16 trials for each point) using a stylus pen as input device. We applied a flexible tracking procedure, as would be realistic during analysis of video data for research purposes. This means a complementary application of both tracking modes (progressive or frame-wise), adjustment of view (zoom) and playback speed, and correction of tracking, if required. The goal was to maximize both tracking speed *S* and tracking precision *P*, i.e. use progressive mode where possible and frame-wise mode where necessary. Precision *P* for x- and y-coordinates was calculated as(7)
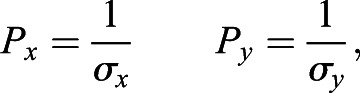
with *σ*_*x*_ and *σ*_*y*_ being the standard deviation of the x- and y-coordinates, respectively. Note that, in contrast to accuracy *A* (the closeness of tracked points to a known reference point), precision *P* quantifies the closeness of the tracking trials to each other, being a measure for reproducibility. For each point in the video, we measured the average tracking speed *S*, the relative amount of progressive tracking used, and the tracking precision *P*_*x*_ and *P*_*y*_.

## Supplementary Material

Supplementary information

## References

[BIO055962C1] Brainerd E. L., Baier D. B., Gatesy S. M., Hedrick T. L., Metzger K. A., Gilbert S. L. and Crisco J. J (2010). X-ray reconstruction of moving morphology (XROMM): precision, accuracy and applications in comparative biomechanics research. *J. Exp. Biol.* 313, 262-279. 10.1002/jez.589 20095029

[BIO055962C2] Brown D. and Cox A. J (2009). Innovative uses of video analysis. *Phys. Teach.* 47, 145-150. 10.1119/1.3081296

[BIO055962C3] Crall J. D., Gravish N., Mountcastle A. M. and Combes S. A (2015). BEEtag: a low-cost, image-based tracking system for the study of animal behavior and locomotion. *PLoS ONE* 10, e0136487 10.1371/journal.pone.0136487 26332211 PMC4558030

[BIO055962C4] Druelle F., Aerts P., Ngawolo J. C. B. and Narat V (2020). Impressive arboreal gap-crossing behaviors in wild bonobos, *Pan paniscus*. *Int. J. Primatol.* 41, 129-140. 10.1007/s10764-020-00140-z

[BIO055962C5] Fagarasanu M. and Kumar S (2003). Carpal tunnel syndrome due to keyboarding and mouse tasks: a review. *Int. J. Ind. Ergon.* 31, 119-136. 10.1016/S0169-8141(02)00180-4

[BIO055962C6] Hedrick T. L. (2008). Software techniques for two-and three-dimensional kinematic measurements of biological and biomimetic systems. *Bioinsp. Biomim.* 3, 034001 10.1088/1748-3182/3/3/034001 18591738

[BIO055962C7] Karlqvist L. K., Hagberg M., Köster M., Wenemark M. and Anell R (1996). Musculoskeletal symptoms among computer assisted design (CAD) operators and evaluation of a self-assessment questionnaire. *Int. J. Occup. Environ. Health.* 2, 185-194. 10.1179/oeh.1996.2.3.185 9933873

[BIO055962C8] Knörlein B. J., Baier D. B., Gatesy S. M., Laurence-Chasen J. D. and Brainerd E. L (2016). Validation of XMALab software for marker-based XROMM. *J. Exp. Biol.* 219, 3701-3711. 10.1242/jeb.145383 27655556

[BIO055962C9] Kotani K. and Horii K (2003). An analysis of muscular load and performance in using a pen-tablet system. *J. Physiol. Anthropol. Appl. Hum. Sci.* 22, 89-95. 10.2114/jpa.22.89 12672972

[BIO055962C10] McElreath R. (2020). *Statistical Rethinking: A Bayesian Course with Examples in R and Stan*, 2nd edn, CRC press.

[BIO055962C11] Mielke F., Van Ginneken C. and Aerts P (2020). Quantifying intralimb coordination of terrestrial ungulates with Fourier Coefficient Affine Superimposition. *Zool. J. Linn. Soc.* 189, 1067-1083. 10.1093/zoolinnean/zlz135

[BIO055962C12] Python Software Foundation (2020). Python Language Reference, version 3.8. Available at http://www.python.org.

[BIO055962C13] R Core Team (2020). *R: A Language and Environment for Statistical Computing*. Vienna, Austria: R Foundation for Statistical Computing https://www.R-project.org.

[BIO055962C14] Salvatier J., Wiecki T. V. and Fonnesbeck C (2016). Probabilistic programming in Python using PyMC3. *PeerJ Comput. Sci.* 2, e55 10.7717/peerj-cs.55 PMC10495961 37705656

[BIO055962C15] Ullman J., Kangas N., Ullman P., Wartenberg F. and Ericson M (2003). A new approach to the mouse arm syndrome. *Int. J. Occup. Saf. Ergon.* 9, 463-477. 10.1080/10803548.2003.11076583 14675519

