# Supplementary material for: Progressive tracking: a novel procedure to facilitate manual digitization of videos
Source: Biol Open. 2020 Nov 6;9(11):bio055962. doi: 10.1242/bio.055962 (PMC7657473; doi:10.1242/bio.055962)
Supplement: Supplementary information [file biolopen-9-055962-s1.pdf]

File S1

Click here to Download File S1

File S2

Click here to Download File S2

File S3

Click here to Download File S3

File S4

Click here to Download File S4

Biology Open • Supplementary information

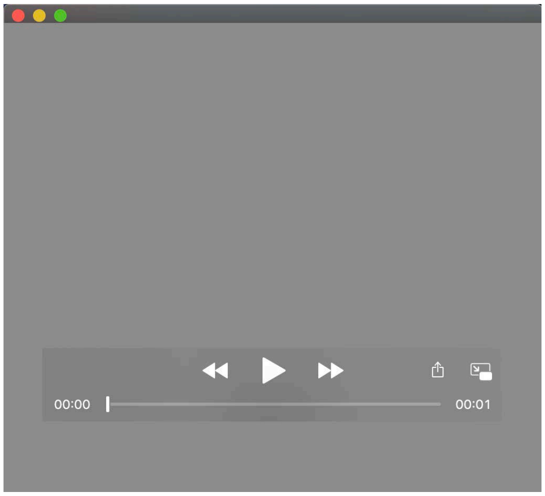

Movie 1. Tracked Piglet Movie

Table S1

Click here to Download Table S1

Table S2

Click here to Download Table S2

Biology Open • Supplementary information
